# Are medical school preclinical tests biased for sex and race? A differential item functioning analysis

**DOI:** 10.1186/s12909-024-06540-6

**Published:** 2025-01-29

**Authors:** Esther Dasari Dale, Mohammed A. A. Abulela, Hao Jia, Claudio Violato

**Affiliations:** 1 University of Minnesota Medical School, 420 Delaware Street SE, Mayo Building, Minneapolis, MN 55455 USA; 2 Department of Educational Psychology, University of Minnesota, Minneapolis, MN 55455 USA; 3 Associate Professor of Educational Psychology, South Valley University, Qena, Egypt

**Keywords:** Differential item functioning, Test validity, Race and sex bias, Psychometric analysis

## Abstract

**Background:**

A common practice in assessment development, fundamental for fairness and consequently the validity of test score interpretations and uses, is to ascertain whether test items function equally across test-taker groups. Accordingly, we conducted differential item functioning (DIF) analysis, a psychometric procedure for detecting potential item bias, for three preclinical medical school foundational courses based on students’ sex and race.

**Methods:**

The sample included 520, 519, and 344 medical students for anatomy, histology, and physiology, respectively, collected from 2018 to 2020. To conduct DIF analysis, we used the Wald test based on the two-parameter logistic model as utilized in the IRTPRO software.

**Results:**

The three assessments had as many as one-fifth of the items that functioned statistically differentially across one or more of the variables sex and race: 10 out of 49 items (20%), six out of 40 items (15%), 5 out of 45 items (11%) showed statistically significant DIF for *Anatomy*, *Histology*, and *Physiology* courses, respectively. Measurement specialists and subject matter experts independently reviewed the items to identify construct-irrelevant factors as potential sources for DIF as demonstrated in Appendix A. Most identified items were generally poorly written or had unclear images.

**Conclusions:**

The validity of score-based inferences, particularly for group comparisons, requires test items to function equally across test-taker groups. In the present study, we found DIF of some items for sex and race in three content areas. The present approach should be utilized in other medical schools to address the generalizability of the present findings. Item level DIF should also be routinely conducted as part of psychometric analyses for basic sciences courses and other assessments.

**Clinical trial number:**

Not applicable.

**Supplementary Information:**

The online version contains supplementary material available at 10.1186/s12909-024-06540-6.

## Background

An important procedure in test design, development, and validation is to examine whether test items function equally across test-taker groups, which is necessary for fairness of score-based inferences. In more detail, students with equal proficiency score (also called latent ability or theta) should have the same probability of correctly answering a test item regardless of their group membership. If not, this indicates one or more test items likely measure factors other than the construct being assessed (i.e., construct-irrelevant factors), and consequently may favor one group over another. To support the fairness of test scores interpretation and uses, differential item functioning (DIF) has been proposed to examine if one or more test items function differently across test-takers who have equal latent ability [1]. In that sense, DIF occurs when test-takers with the same latent ability but from different groups have an unequal probability of correctly answering a test item.

DIF is, therefore, a psychometric procedure for detecting whether a particular test-taker group, typically the minority or the focal group, performs differently on one or more test items compared to the majority or the reference group conditioned on their latent ability. DIF analyses identify items with different levels of difficulty (uniform DIF) and discrimination (nonuniform DIF) for equal ability test-takers groups (e.g., women or men, race groups, SES, etc.). When this is the case, such items are likely to favor the reference group, leading to potential bias and consequently fairness issues. Specifically, bias at the item-level undermines the validity of score interpretations and consequently the fairness of the intended score use [1]. This is especially true in high-stakes testing such as summative examinations in foundational sciences courses, which can eventually lead to adverse results on these examinations for the focal group.

Accordingly, DIF has been widely used in testing programs, particularly in STEM (Science, Technology, Engineering, and Mathematics); however, DIF has not been commonly used in medical education [2]. Due to the paucity of DIF analysis employed in medical education, particularly for foundational courses, the main purpose of the present study was to extend DIF analyses to basic sciences courses in the preclinical medical curriculum. This has the potential to enhance equity and fairness in assessment practices in medical education.

The preclinical medical education program traditionally emphasizes basics sciences relevant to medicine such as anatomy, histology, physiology, pathology, and genetics, among others. Midterm exams, final exams, and other tests are used to assess whether medical students have acquired the fundamentals necessary for the further study of medicine and involvement in patient care. Final and other exam scores are frequently summative resulting in pass/fail or tiered (e.g., honors, excellence, satisfactory, etc.) grading decisions. Such scores are also thought to predict performance on the United States Medical Licensing Examination (USMLE) board exams [3]. Midterms and course exam scores can also provide evidence of teaching efficacy, identifying areas of strength or needed improvements of students (i.e., competency-based medical education) [4].

Researchers have extensively investigated the validity and reliability of medical course examination in various forms, such as paper-and-pencil exams, computer-based exams, and Objective Structured Clinical Examination (OSCEs) [5–8]. In validity studies, researchers and users need to detect if there is test bias, such as whether men and women, who have equal underlying abilities, perform differently or if the performance is linked with test-takers’ race [9]. Attending to the performance of different groups is important for equity – assessments and examinations should not discriminate against any individual or a specific group of test-takers [10]. In medical education, some studies have explored the sex^1^ and/or race differences in test performance [11–16], academic and financial stress [17], medical communication [18], and patient-physician relationship [19]. These studies focused on group differences for macro-level measurements, such as cumulative GPAs, acceptance rates, and total test scores. Based on this review, few studies, to date, have explored the extent of item-level measurement variance (e.g., due to bias) attributable to sex and/or race, particularly for end-of-course assessments as an important operational procedure to ensure fairness of score interpretation and uses. Detecting, editing, and perhaps removing these items improves the validity of test score interpretations and uses. Additionally, item bias (as initially detected by DIF) can be discouraging for students from disadvantaged backgrounds or racial minorities from developing interest in a course subject, and it is therefore desirable to reduce such potential item bias [20].

Each test item should be designed to assess students’ understanding of the construct being measured. For example, items in physiology course exams should measure test-takers’ physiology knowledge and comprehension or application rather than knowledge relating to cultural background or other demographic characteristics. Some items measure irrelevant constructs to varying extent and hence DIF occurs [21, 22], since it indicates construct-irrelevant variance (i.e., variability in scores is due to factors unrelated to what the item is intended to measure). DIF analysis allows us to compare the item-level performance of groups while simultaneously predicting students’ potential to score on the course examinations [23–25]. DIF analysis can also identify achievement gaps that are not revealed when comparing total scores [26]. Thus, identifying DIF items has implications for the enhancement of validity of score interpretations and fairness as well as for evidence-based assessment across sex and racial groups.

Although there is little research within medical education that has found evidence of DIF across sex, other areas such as mathematics achievement testing, have resulted in evidence that sex related DIF remains a large concern [27, 28]. These recent studies have often found inconsistent or even contradictory results because of the possible interaction effects of different cohorts, constructs, and selectivity of the sample [29]. These data suggest that when responding to items that require prior science courses knowledge and spatial cognitive abilities as might be the case in anatomy items. For example, the sex-differences may result in different probabilities of answering correctly although test-takers are of the same ability level.

The issue of race-based differences in achievement has generated much attention, controversy and debate since at least the 1960s. The underachievement of racial minorities, as compared to their white peers, has been observed in different contexts [30]. For medical school acceptance rates, for example, Asian Americans and Whites are proportionately overrepresented in medicine [31]. Moreover, Black and Latino test-takers’ mean Medical College Admission Test (MCAT) scores are lower than White candidates’, mirroring differences on other standardized admission tests and in the average undergraduate grades of medical school applicants [14].

A British study also found that trained doctors and medical students from minority ethnic groups tended to underperform academically compared with their White counterparts; the authors concluded such differences are unlikely to be primarily caused by examiner bias or candidate communication skills [15]. Other studies have shown significant race-based differences in motivation [16] and distress [32]. These foregoing findings have implications for explaining the test score differences for preclinical exams because of the variance producing potential irrelevant factors [21] such as sex and race, which may lead to different response behaviors even within the same ability levels. Taken together, differences in test scores should be investigated whether being actual differences in latent ability or attributed to construct-irrelevant factors, potentially detected by conducting DIF.

## Study rationale

One of the recommended assessment practices is to investigate the presence of construct-irrelevant variance in preclinical foundational medical sciences course exams, which likely leads to potential bias. There is currently little published work that has addressed the issue of potential test item bias in preclinical sciences course exams using advanced psychometric techniques such as DIF based on item response theory (IRT), which is a robust measurement framework commonly employed in high-stakes settings (e.g., certification and licensure boards exams).

The main purpose of the present study, therefore, was to extend the implementation of DIF analyses to selected content, such as *Anatomy*, *Histology*, and *Physiology* course exams in a medical schools’ first and second preclinical years. It is essential to explore items to see if DIF exists based on sex and race groups. Reviewing items showing DIF may provide insights as to why such items are behaving this way. The present study can inform assessment practices by establishing evidence-based guidelines for the importance of applying DIF in foundational sciences courses, as well as other assessments.

## Methods

### Participants

Data were collected from medical students who reported their sex and race as part of the demographics during the test administration sessions at a Medical School in a large Midwestern Public University. Table 1 shows the total number of students who were in *Anatomy* (*n* = 520), *Histology* (*n* = 519), and *Physiology* (*n* = 344) courses in the Fall 2018, Fall 2019, and Fall 2020. Some students did not report their race during the test administration session. Data based on sex was investigated as males vs. females, where race data was studied between white vs. non-white, since some racial groups (e.g., African Americans) were too small to conduct the analysis and yield robust results, particularly with the adopted IRT framework.

**Table 1:** Undergraduate medical students in anatomy, histology, and physiology according to their sex and race

Preclinical Sciences Courses	Demographic variables				
	Sex			Race	
	Males *n* (%)	Females *n* (%)		White *n* (%)	Non-white *n* (%)
*Anatomy*	228 (44%)	292 (56%)		323 (65%)	175 (35%)
Total N	520			498	
*Histology*	227 (44%)	292 (56%)		325 (65%)	172 (35%)
Total N	519			497	
*Physiology*	198 (56%)	146 (44%)		214 (63%)	125 (37%)
Total N	344			339	

For Anatomy and Histology, the non-White group included 7 American Indian or Alaska Native students, 76 Asian students, 47 Black or African American students, 10 Native Hawaiian or Other Pacific Islander students, and 17 students identified as ‘Other.’ Additionally, 18 students did not respond to the race question (note that only 15 students did not respond to the race question for Histology). For Physiology, among the 125 non-White students, there were 5 American Indian or Alaska Native students, 59 Asian students, 31 Black or African American students, 7 Native Hawaiian or Other Pacific Islander students, 13 students identified as ‘Other,’ and 10 students who did not respond to the race question.

### Assessment of preclinical sciences

To enhance validity of score interpretations, the *Office of Assessment and Evaluation* works with faculty to improve their item writing to minimize construct-irrelevant sources of variance. For operational purposes, each test as well as the individual items are analyzed for measurement quality. Item analysis examines individual medical student responses to the test to ensure the quality of scored items and to remove ambiguous, misleading, or miskeyed items. The three science courses are described below.

#### The anatomy test

*Anatomy* is offered in the first semester and the current items are drawn from the midterm exams comprising 85 multiple-choice items (MCIs) with a time limit of two hours. Content is drawn from the first half of the semester and focuses on extremities, the back, and the thorax. The content is aligned with the intended learning outcomes described in the syllabus. Out of the 85 items, 49 items that appeared in three exams were analyzed.

#### The histology test

Similar to Anatomy, Histology is offered in the first semester and the test has 40 MCIs completed over a period of two hours. Content is drawn from the first half of the semester and focuses on epithelia, connective tissue, skin, muscle, nerve, bone, osteogenesis, blood, and the cardiovascular system.

#### The physiology test

The *Physiology* course is offered in the second semester and the test has 60 MCIs completed in two hours. The content is drawn from the first half of the semester and focuses on skeletal muscle and cardiovascular physiology including the topics of cardiac action potentials, cardiac conduction systems, cardiac output, cardiac cycle, capillary filtration, regulation of venous return, electrocardiograms, local control and endocrine control of circulation, circulation, blood pressure and exercise, hemodynamics, echocardiography, compliance recoil, surfactant, airway resistance, alveolar ventilation, partial pressure, gas exchange, pulmonary circulation, O2 and CO2 transport, ventilation perfusion matching, pulmonary function testing, neural and chemical regulation of breathing, and pulmonary fibrosis.

### Procedures

A careful comparison of the three test item sets over the three years (Fall 2018 - Fall 2020) showed that the item stem and options were identical for each year that they were administered. We employed computer-based testing software for a stable testing environment and all midterm and final exams are administered under standard conditions. The digital platform allows the delivery of secure exams without depending on a constant internet connection. Students must use a unique password to login on the test, which is provided five minutes before the start of the exam. For security purposes, a lockdown browser prevents students from copying, printing, or visiting other applications or websites during the testing session. A typical midterm and final contains 75 items that need to be answered within two hours.

### Data Analysis

In addition to overall item analyses, we conducted DIF analyses to determine if test items function equally across student groups. DIF methods rely on statistical significant testing to identify items that function differently across test-taker groups. Specifically, we utilized IRT-based procedures because they have been found to be robust to type I errors (i.e., false positives) using IRTPRO software [33] based on the two-parameter logistic model (2PL Model; item difficulty and item discrimination), and model-data fit was evaluated using the commonly utilized fit indices. In the current study, we conducted DIF using the Wald test with item parameter error variance-covariance matrices computed using the supplemental expectation–maximization (SEM), an algorithm employing an iterative method to find maximum likelihood estimates of parameters in statistical models, where the model depends on unobserved latent variables [34]. To determine anchor items, we utilized the default option in IRTPRO, “*Test all items*,* anchor all items*”, which produced three tables: two for item parameters for each group and a third for the initial DIF results. Next, items that were not statistically significant were used as anchors, and we re-ran the analysis using the third option, “*Test candidate items*,* estimate group differences with anchor items*”, to identify the final statistically flagged items.

To statistically flag items, IRTPRO provides three values for the χ^2^ statistic with their associated degrees of freedom and *p* values for each item in the test; one for both parameters (*a* and *b*, nonuniform DIF), one for the discrimination parameter (*a*,* n*onuniform DIF), and the other for the difficulty parameter (*b*, uniform DIF). To elaborate, uniform DIF occurs when a particular test-taker group (e.g., the reference group) has a higher ability of correctly answering the studied item across the entire ability continuum. Conversely, nonuniform DIF occurs when the probability of correctly answering the items differs across test-taker groups, with one group (usually the focal group) having higher probability at the lower ability continuum, but another group has a greater probability at the higher ability continuum [35].

The null hypothesis tested by the total χ^2^ statistic is that “both *a* and *b* parameters are equal across groups”. It has two degrees of freedom because it is a test statistic for the two parameters: discrimination and difficulty. The null hypothesis tested by the χ^2^ statistic for the discrimination parameter is that “the *a* parameter is equal across groups”. It has one degree of freedom because it is a test statistic only for the discrimination parameter. The null hypothesis tested by the χ^2^ statistic for the difficulty parameter is that “the *b* parameter is equal across groups”. It has one degree of freedom because it is a test statistic only for the difficulty parameter.

There are three stages to determine which item has a significant DIF: (1) determine if there is significant DIF for discrimination, difficulty or both parameters; (2) determine if discrimination only is significant which means the item discriminates differently across the latent trait or has nonuniform DIF; and (3) determine if only the difficulty parameter is significant suggesting the item has uniform DIF. There is also a special type of nonuniform DIF when both the difficulty and discrimination parameters are significant. The significance level, adopted in the current study to flag items, is *p* < .05, which is commonly utilized in the DIF literature. We opted for that liberal significance threshold for not missing items that are likely to possess DIF. Additionally, for DIF analyses, the cost of Type II error (i.e., classifying items that function differently as DIF-free) is more than that of Type I error (i.e., classifying a DIF-free item as possessing DIF). The former causes fairness issues for the focal group, while the latter adds additional effort in the sensitivity analyses.

It is important to note that DIF and item bias are distinct concepts. DIF provides statistical evidence suggesting the possibility of bias. However, items identified as having DIF are subsequently reviewed by experts to determine whether they are actually biased. Stated differently, items statistically flagged as possessing DIF should go through another stage of sensitivity analysis by measurement specialists and SMEs to respectively identify violations of item writing guidelines and inappropriate content [36]. Thus, DIF is a psychometric/statistical significance testing procedure followed by sensitivity analysis to conclude the potential for item bias.

## Results

Prior to presenting DIF results, the 2PL model-data fit is presented in Table 2.

**Table 2:** The 2PL model-data fit indices for anatomy, histology, and physiology

Course	M2	df	CFI	TLI	SRMSR	RMSEA	95% CI	
Anatomy	1766^***^	1127	0.88	0.87	0.066	0.041	0.037	0.044
Histology	762	740	0.99	0.98	0.055	0.007	0	0.015
Physiology	985	945	0.96	0.96	0.061	0.011	0	0.019

Note. CI = confidence intervals, df = degrees of freedom, CFI = comparative fit index, TLI = Tucker-Lewis index, RMESA = root mean square error of approximation, SRMSR = standardized root mean squared residual

Based on Table 2, Histology and Physiology had excellent model-data fit, since the M2 statistic was not significant, the CFI and TLI indices were above 0.90, and the RMSEA and SRMSR were below 0.08. For anatomy, despite the CFI and TLI indices were relatively lower than the recommended threshold, the RMSEA and SRMSR were very good indicating model-data fit.

Results for the flagged items are presented below for the three preclinical sciences courses: (a) Anatomy (Table 3), (b) Histology (Table 4), and (c) Physiology (Table 5) for two studied variables: (a) sex and (b) race.

### Anatomy

As shown in Table 3, 10 out of 49 items (approximately 20%) showed statistically significant DIF at least in one of the two studied variables. Specifically, items 20 and 32 showed DIF for the two studied variables. For sex, the χ^2^ statistic was significant for seven items indicating that 14% of the anatomy test items showed statistically significant DIF, favoring male students. For race, five items (approximately 10%) had statistically significant DIF, favoring white students.

**Table 3:** DIF statistics for anatomy for sex (males vs. females; *N* = 520) and race (white vs. nonwhite; *N* = 498)

Variable	Item	Total (*df* = 2)		Discrimination (*df* = 1)		Difficulty (*df* = 1)		DIF Type
		χ^2^	*p*	χ^2^	*p*	χ^2^	*p*	
Sex	16	8.3	0.016	7.6	0.006	0.7	0.420	Nonuniform
	17	8.8	0.012	6.6	0.010	2.2	0.141	Nonuniform
	20	6.5	0.038	4.8	0.028	1.7	0.194	Nonuniform
	22	6.9	0.032	0.4	0.504	6.5	0.011	Uniform
	32	6.2	0.046	0.5	0.495	5.7	0.017	Uniform
	39	8.5	0.014	0.6	0.451	8.0	0.005	Uniform
	49	8.5	0.014	5.5	0.019	3.0	0.085	Nonuniform
Race	20	6.4	0.040	6.0	0.014	0.4	0.506	Nonuniform
	30	9.6	0.008	3.4	0.066	6.2	0.013	Uniform
	32	7.0	0.030	7.0	0.008	0.0	0.878	Nonuniform
	41	7.2	0.027	6.4	0.011	0.8	0.359	Nonuniform
	47	6.2	0.046	5.2	0.023	1.0	0.317	Nonuniform

### Histology

As contained in Table 4, six out of 40 items (15%) showed statistically significant DIF at least in one of the two studied variables. Specifically, item 27 showed DIF for the two studied variables. The number of items flagged as possessing DIF based on sex was fewer than *Anatomy*; the χ^2^ statistic was significant for only two items (5%), which favored male students. Five items (approximately 13%) demonstrated statistically significant DIF for race, favoring white students.

**Table 4:** DIF statistics for histology for sex (males vs. females; *N* = 519) and race (white vs. nonwhite; *N* = 497)

Variables	Items	Total (*df* = 2)		Discrimination (*df* = 1)		Difficulty (*df* = 1)		DIF Type
		χ^2^	*p*	χ^2^	*p*	χ^2^	*p*	
Sex	27	11.0	0.004	10.9	0.001	0.1	0.784	Nonuniform
	30	91.4	0.001	9.9	0.002	81.5	0.001	Both
Race	2	71.1	0.001	26.1	0.001	45.0	0.001	Both
	10	9.7	0.008	0.3	0.591	9.4	0.002	Uniform
	15	9.3	0.009	2.4	0.125	7.0	0.008	Uniform
	27	117.0	0.001	30.2	0.001	86.8	0.001	Both
	34	9.7	0.008	0.4	0.551	9.3	0.002	Uniform

Note. Both refers to a type of nonuniform DIF where the χ^2^ statistic is significant for both the difficulty and discrimination parameters

### Physiology

As shown in Table 5, five out of 45 items (approximately 11%) showed statistically significant DIF at least in one of the two studied variables. For sex, three items (7%) showed statistically significant DIF, favoring males. Only two (4%) items showed statistically significant DIF for race, favoring white students.

**Table 5:** DIF statistics for physiology for sex (males vs. females; *N* = 344) and race (white vs. nonwhite; *N* = 339)

Variables	Item	Total (df = 2)		Discrimination (df = 1)		Difficulty (df = 1)		DIF Type
		χ^2^	*p*	χ^2^	*p*	χ^2^	*p*	
Sex	22	8.8	0.013	4.8	0.028	3.9	0.047	Both
	27	6.3	0.044	0.1	0.771	6.2	0.013	Uniform
	28	8.0	0.019	2.3	0.130	5.7	0.017	Uniform
Race	10	8.5	0.014	0.0	0.919	8.5	0.004	Uniform
	32	9.6	0.009	6.2	0.013	3.5	0.063	Nonuniform

Note. Both refers to a type of nonuniform DIF where the χ^2^ statistic is significant for both the difficulty and discrimination parameters

## Discussion

The overall objective of the present study was to extend the implementation of DIF analyses to medical assessments, particularly for preclinical courses to detect potentially biased items. Overall, 10 out of 49 items (20%), 6 out of 40 items (15%), and 5 out of 45 items (11%) showed statistically significant DIF for *Anatomy*, *Histology*, and *Physiology*, respectively. However, when items are statistically identified as showing DIF, sensitivity reviews were conducted to discern the sources of construct-irrelevant variance as a potential factor leading to DIF. Potential factors underlying DIF might be related to item content and/or violations of item writing guidelines. Appendix A contains examples of an in-depth sensitivity analysis of the potential reasons for which some items were identified as possessing DIF.

Overall, a potential reason for which performance varied with sex for *Anatomy* items may be related to sex differences in spatial ability. In their extensive review and studies, Maccoby and Jacklin [37] concluded that in the cognitive domain, men outperformed women in quantitative and visual-spatial areas, whereas women outperformed men in verbal ability. Additionally, Maeda and Yoon [38] in a meta-analysis of 3-D mental rotation ability using the Purdue Spatial Visualization Tests: Visualization of Rotations (PSVT: R) integrated 70 effect sizes of gender differences in 40 primary studies. Results indicated that male test-takers outperformed their female peers. In a meta-analysis focused specifically on the teaching and testing of anatomy, Yammine and Violato [39] also found spatial-relational differences in men and women medical students as have other researchers [40].

Regarding *Histology*, one possible reason performance varied with sex for some histology items may also be the use of images. In several studies, students were asked about reasons for which they had problems interpreting histological images: 77% stated that their difficulty in interpreting histology images was due to their lack of knowledge of anatomy, followed by their difficulty in delimiting cells (72%), and histological sections orientation (62%) [10, 11].

For *Physiology*, another possible reason performance varied with sex on physiology items could relate to the use of images and sex differences in the other science disciplines that underpin physiology. The use of images is a key component teaching and testing in *Anatomy*, *Histology*, and *Physiology*. Poor quality images may confuse students and over-demand spatial-relational processing, resulting in confused responses, potentially for focal group students.

From an assessment perspective for both sex and race, many items that demonstrated DIF had short, incomplete or vague stems. Because these items were not in a full interrogative form, some students from a specific group (e.g., non-white) may have been confused at the construct being assessed by the item based on a sentence fragment. Other items had options of unequal length. Readability issues emerged both in the text and the images attached to some of those items. Some items were unnecessarily wordy whereas other items contained superfluous information, all factors of construct-irrelevant variance. Linguistic complexity was another concern that might favor a specific group (e.g., women) over another (men). Similarly, the use of concepts or language expressions that might differ in their meanings across groups, especially race, is a potential source of DIF. These items may include content that is sexist, racist, stereotypic, or specialized to particular contexts. The items in Appendix A exemplify possible reasons underlying DIF according to the sensitivity analysis results conducted by SMEs and measurement specialists.

## Limitations

The present study has some limitations. For instance, we only conducted DIF for selected content areas (anatomy, physiology, histology), at a single medical school. Future work can be expanded to other content areas (e.g., biochemistry, genetics, etc.) and institutions. Additionally, we combined all the nonwhite subgroups (e.g., Asian) into one group due to limited sample size in each of these other subgroups. Future researchers can conduct DIF on each of these other groups when the sample size is large enough to ensure valid DIF results. Last, we only utilized one method to conduct DIF. In future research, more than one method can be used and results can be compared accordingly.

## Conclusion

The validity of score-based inferences requires test items to function equally across groups. In the present study, we found DIF of some items for sex and race in three content areas. DIF was found on some items for both sex and race possibly due, in part, to item flaws and/or poor images. Results from the present study highlighted the importance of performing item-level invariance analysis to ensure validity and fairness of test score interpretations and uses and promote evidence-based assessment practices, particularly in the context of high-stakes assessments in medical education. Therefore, test developers (e.g., course directors and instructors) should have formal training in item and test development and validation. To conclude, item-level DIF should also be routinely conducted as part of psychometric analyses for basic sciences courses and other assessments to ensure fairness of score interpretation and uses.

## Electronic supplementary material

Below is the link to the electronic supplementary material.

Supplementary Material 1

## Data Availability

The datasets analyzed during the current study are available from the corresponding author on reasonable request.

## Abbreviations

DIF: Differential item functioning
IRT: Item response theory

## Footnotes

1 For the present study, we need to clarify the concept of gender and sex because sex is a central variable in differential item functioning. We use gender here as the American Psychological Association (APA) defines it: “the condition of being male, female, or neuter,” referring “especially to social or cultural traits,” in contrast to sex, which the APA defines as “referring especially to physical and biological traits.” Guidelines for psychological practice with lesbian, gay, and bisexual clients. In: *APA Dictionary of Psychology* American Psychological Association. 2015.
