# Supplementary material for: Are medical school preclinical tests biased for sex and race? A differential item functioning analysis
Source: BMC Med Educ. 2025 Jan 29;25:146. doi: 10.1186/s12909-024-06540-6 (PMC11780802; doi:10.1186/s12909-024-06540-6)
Supplement: Supplementary file 1 — Supplementary Material 1 [file 12909_2024_6540_MOESM1_ESM.docx]

**Appendix A**

**Sample Flagged Items and Potential Reasons Underlying DIF**

| **Item** | **Item content** | **Image (if any)** | **Grouping variable** | **Potential reasons underlying DIF** |  |
| --- | --- | --- | --- | --- | --- |
| **Anatomy** | | | | | |
| 16 | Which of the following nerves provides cutaneous innervation to the area indicated area in the attached figure?  The figure is a posterior view of arm, forearm and hand.   1. Lateral (antebrachial) cutaneous nerve of forearm 2. Superficial radial nerve 3. Medial (antebrachial) cutaneous nerve of forearm 4. Ulnar nerve 5. Median nerve | 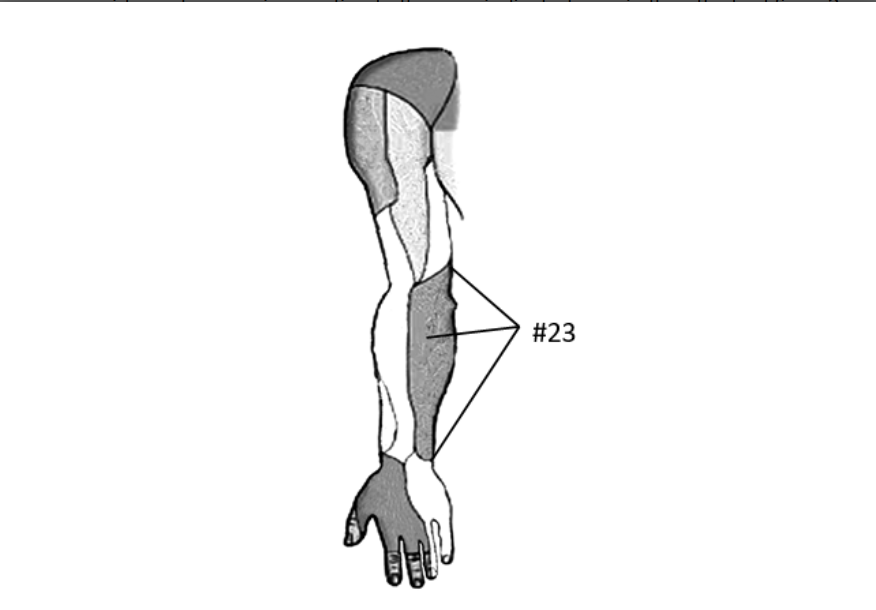 | Sex | The low quality image and potential differences in spatial ability (i.e., a construct-irrelevant factor) between men and women rather than what is the item assessing in anatomy. |  |
| 32 | You are examining a young girl with complaints about an awkward gait. When completing her medical history, she describes that she recently had an intramuscular injection for steroid therapy. From the girl’s description, it sounds like the needle passed into the dangerous superior medial quadrant of her gluteal region.  Upon examination from the posterior, it is obvious that the level of her left iliac crest is lower than the right.  Watching her walk, the level of the left iliac crest does not elevate in order to allow her left foot to swing forward. To compensate, she swings the foot out laterally.  What is the spinal cord contribution of this nerve?  A. Cervical  B. Thoracolumbar  C. Lumbosacral |  | Race | It appears from the question stem that linguistic complexity is likely to be an issue, which could be an issue for the focal group (i.e., non-white) compared to the reference group (i.e., white) |  |
| **Histology** | | | | | |
| 2 | The actin filaments forming the core of microvilli and those connected to the zonula adherens are both linked to the actin filaments of  A. basal bodies  B. the basal lamina  C. the lamina reticularis  D. the terminal web  E. the territorial matrix |  | Race | One of the item writing guidelines likely violated is that four of the five answer options had the definite article “the”, which can cause confusion to specific test-taker group. |  |
| **Physiology** | | | | |  |
| 28 | Use the accompanying diagram (attached) to answer the following question. What is the cellular mechanism responsible for the transition from point 1 to point 2?  A. Intracellular calcium levels increasing above previous systolic levels  B. Recruitment of ventricular cells that depolarize and participate in systole  C. Sarcomeres stretched and arranged at an optimal starting length  D. Stretch and stimulation of atrial baroreceptors | 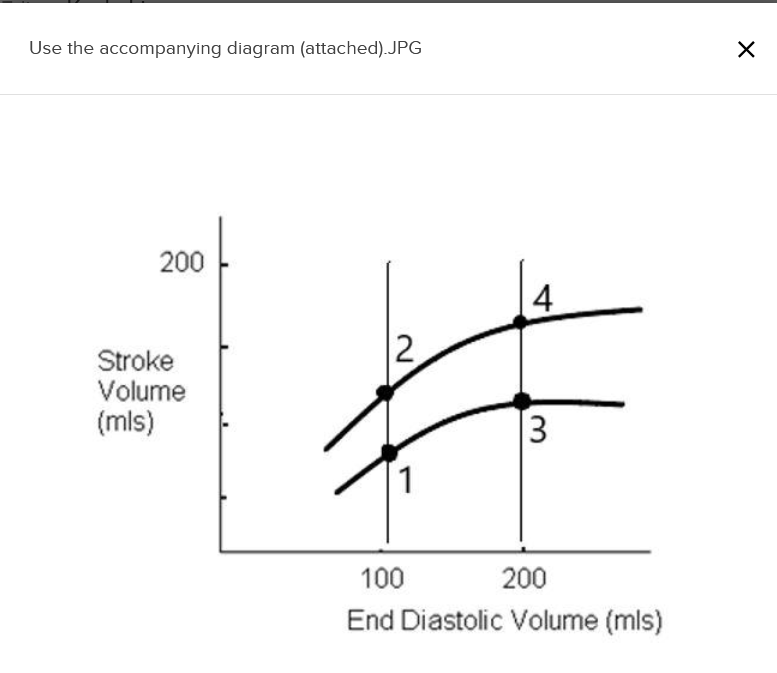 | Sex | Similar to item 16 in “Anatomy”, the low quality image likely makes the item more difficult for women compared to men. |  |
| 10 | Higher than resting circulating epinephrine levels most likely cause what choice below?  A. Decreased cardiac output due to both a fall in heart rate and a fall in stroke volume  B. Decreased activity of the Ca2+ ATPase in the myocardial sarcoplasmic reticulum  C. Increased duration of ventricular systole  D. Increased vasodilation in skeletal muscle |  | Race | It is noted that two out of the four answer options has shorter phrasing, which is a clear violations of item writing guidelines. |  |
